# The Use and Outcomes of Compassion‐Focused Group Interventions With Children and Adolescent Clinical Populations: A Systematic Review and Narrative Synthesis

**DOI:** 10.1002/cpp.70259

**Published:** 2026-03-22

**Authors:** Georgia Roberts, Nicole Parish, Victoria Samuel

**Affiliations:** ^1^ South Wales Doctoral Programme in Clinical Psychology, 11th Floor Tower Building Cardiff University Cardiff UK

**Keywords:** adolescent mental health, child mental health, compassion‐focused therapy, compassion‐focused therapy groups, systematic review, third wave

## Abstract

Research has investigated the outcomes of compassion‐focused therapy for adult populations, with systematic reviews and meta‐analyses supporting its effectiveness. Comparatively, the literature investigating compassion‐focused interventions for child and adolescent populations is less developed, with fewer empirical studies to date. Most existing literature on compassion interventions has been in a group context. A systematic review was conducted to explore the state of the evidence base for compassion‐focused groups used with child and adolescent clinical populations. This narrative synthesis aimed to review the methodological quality and outcomes of the published and unpublished literature. Ovid, Scopus, EBSCO and ProQuest platforms were utilised to search databases with studies from the year 2005 onwards. Nine studies were identified, including 138 participants receiving a compassion‐focused intervention aged between 11 and 17 years. There was some evidence to suggest that compassion‐focused groups have the potential to increase self‐compassion and improve the wellbeing of children and adolescents. However, the outcomes were varied, and methodological concerns limited interpretations of results. Additionally, the heterogeneity between studies made it challenging to synthesise the literature and draw conclusions regarding the effectiveness of these groups for this population. Future research would benefit from higher quality empirical studies comparing compassion‐focused groups to other interventions, alongside a greater consistency in valid outcome measure use across research.

## Abstract (highlights)

Summary

- This review considers the use and outcomes of compassion‐focused group interventions as a transdiagnostic approach for children and adolescents.
- Group interventions are a common method of therapy delivery and therefore, a review of these for this population has implications for clinical practice.
- There was some preliminary evidence that compassion‐focused group interventions can improve both compassion‐focused and wider psychological outcomes. However, the outcomes were mixed, and this review was limited by considerable heterogeneity in study design, outcome measures used and samples recruited.
- All the studies included were very recent (from 2020 onwards). Therefore, this study has reviewed an emerging body of evidence, and consequently, learning from the limitations may help to guide future clinical practice and research in this area.

## 1 Introduction

### 1.1 Compassion‐Focused Therapy

Compassion‐focused therapy (CFT) is an integrated approach, drawing on ideas from evolution, social, developmental and Buddhist psychology, attachment theory and neuroscience (Gilbert 2009). CFT conceptualises mental health difficulties as being rooted in patterns of self‐relating, with shame and self‐criticism playing a central role (Gilbert and Irons 2005). CFT is a therapeutic intervention which aims to improve psychological wellbeing by helping individuals to have a compassionate relationship with themselves and others (Gilbert 2010b). Compassion is defined by Gilbert (2014, 19) as ‘a sensitivity to suffering in self and others, with a commitment to try to alleviate and prevent it’. Within this CFT intervention, individuals are supported to enhance experiences of compassion towards themselves, towards others, and feel more comfortable receiving compassion from others (Gilbert 2014).

CFT conceptualises emotion regulation in terms of three affect regulation systems which have evolved to organise human beings motivational, emotional and physiological responses: the threat protection system; the drive, resource‐seeking and excitement system; and the soothing and safeness system (Gilbert 2009, 2014). Psychological distress is understood to arise when these three systems become functionally unbalanced, for example, when shame and self‐criticism chronically activates the threat system (Gilbert 2020). Such imbalances may stem from early relational experiences, as caring strategies—towards oneself, towards others and in receiving care—often fail to develop in the context of hostile, unpredictable or neglectful early attachment relationships (Gilbert 2020). Consequently, a central aim of CFT interventions is to support individuals' ability to experience a sense of soothing and safety, through cultivating compassion in relationships to their self and others (Gilbert 2009).

Compassionate mind training (CMT) is a CFT intervention which aims to reduce psychological distress through enhancing a greater sense of compassion. Research has found that people may experience ‘blocks’ or ‘fears’ of compassion (Gilbert et al. 2011). Compassion may be viewed as weak, distressing, self‐indulgent or not deserved (Gilbert 2009; Gilbert and Mascaro 2017). CMT addresses these fears, starting with psychoeducation around the ‘tricky brain’ and how it is ‘not our fault’ that our brain responds the way it does (Gilbert 2017). CMT also identifies safety strategies that people use to avoid painful emotions and supports them to overcome these by bringing compassion to the individual's self‐critical thoughts and behaviours (Gilbert and Procter 2006). Additionally, CMT supports people to activate their soothing system through experiential exercises like compassionate imagery, chair work, breathing, compassionate thinking and compassionate letter writing (Gilbert and Procter 2006).

### 1.2 Other Models of Compassion

There are different theoretical and therapeutic approaches to cultivating compassion. One of the most influential is K. Neff's (2003a) model of self‐compassion, which comprises three core components: self‐kindness (versus self‐judgement), common humanity (versus isolation) and mindfulness (versus over‐identification). This model led to the development of the self‐compassion scale (SCS; K. D. Neff 2003b), a widely used self‐report measure assessing how often people engage in various thoughts, feelings and behaviours related to these components. Building on this, Germer and Neff (2019) developed Mindful Self‐Compassion (MSC), an 8‐week mindfulness‐based self‐compassion training program incorporating written exercises, meditation and informal practices that integrate self‐compassion into daily life. While both MSC and CFT aim to enhance compassion, MSC is a skills‐based program often used for general wellbeing, whereas CFT is a broader psychotherapy model more established within clinical services. Therefore, this review will predominantly focus on CFT as the primary theoretical model underpinning compassion‐focused interventions. However, wider compassion‐focused interventions will also be explored if used with clinical populations.

### 1.3 Children and Adolescents

There are many important, and at times difficult, transitions in childhood and adolescence, including physiological, social and interpersonal changes (Gilbert and Irons 2005). CFT, grounded in evolutionary theory, proposes that as young people mature, social motivational systems develop, increasing sensitivity to acceptance and concern over how others perceive them (Gilbert and Irons 2009). In addition, cognitive abilities develop throughout early life (Moshman 2020), which may facilitate engaging in self‐compassion but also can make young people vulnerable to difficulties such as self‐consciousness, self‐criticism and shame (Volkaert et al. 2022). Children and adolescents may also be subjected to experiences of bullying, peer rejection and difficulties within their attachment relationships (Gilbert and Irons 2009). These early experiences can enhance shame and consequently interfere with the fundamental human motivation to be valued, loved and included (Gilbert and Irons 2009). For children and adolescents, when overcoming shame, criticism and avoidance through compassion, Carona et al. (2017) argue that CFT should be adapted to focus not only on the young person, but their specific needs within their interpersonal context, as home, school, culture, community and peer groups may be a possible source of criticism and distress.

Children and young people may also experience ‘blocks’ to self‐compassion. Research found that when interviewing young people, 95% of the sample identified self‐criticism as a barrier to compassion, fearing it would impact their achievements and goal performance (Egan et al. 2022). Some of these young people also described compassion as weak, lazy, and associated it with a sense of stigma (Egan et al. 2022).

### 1.4 Evidence‐Base

Recent reviews have investigated the effectiveness of CFT with adult clinical populations. Craig et al. (2020) reviewed 29 studies: 9 randomised controlled trials (RCTs), 3 controlled trials and 17 observational studies. They found preliminary evidence that CFT may enhance compassion and may contribute to improvements in mental health across various adult clinical populations. In addition, they found that CFT in a group format was the most common and effective format of therapy delivery. However, this review identified significant variability in intervention content and delivery, which limited comparability across studies. Additionally, many included studies lacked control groups, making it difficult to attribute outcomes specifically to CFT as effects may be influenced by other variables. Craig et al. (2020) recommended high‐quality RCTs across diverse populations to strengthen the evidence base.

An updated review by Millard et al. (2023) critiqued Craig et al. (2020) vague definition of ‘clinical populations’, stating that this review included a study which had a non‐clinical sample, parents of children with neurodevelopmental conditions. Millard et al. (2023) conducted the first systematic review and meta‐analysis of 15 randomised CFT trials in clinical populations. They found CFT interventions led to improvements in self‐compassion and self‐reassurance, with small‐to‐large effect sizes, alongside reductions in self‐criticism and fears of compassion. They also found modest improvements in anxiety, depression and eating disorder symptomology. This review supports Craig et al. (2020) preliminary findings that CFT increases self‐compassion and reduces clinical symptomology across varied mental health difficulties.

There is preliminary evidence supporting the use of CFT for adults with chronic health conditions. Two recent systematic reviews (Kılıç et al. 2021; Austin et al. 2021) investigated its effectiveness in these populations. Kılıç et al. (2021) found medium to large improvements in compassion and medium reductions in anxiety, depression, stress and sleep problems. They also reported some condition‐specific benefits, including reductions in fatigue and diabetes‐related distress. Austin et al. (2021) found some improvements in depression, anxiety and pain among adults with cancer or persistent pain. However, this evidence base remains in its infancy, limited by small sample sizes, short‐term follow‐up and limited scope for meta‐analysis due to heterogeneity in outcome measures. Notably, no specific systematic reviews have explored the effectiveness of CFT for children and adolescents with health conditions.

Recently, Perkins et al. (2023) broadly investigated the effectiveness of third‐wave therapies (newer psychological approaches which build on traditional cognitive behavioural therapies) for children and adolescents. This meta‐analysis reviewed 50 RCTs, and two of these studies investigated the effectiveness of CFT. They included both mental and physical health populations. They aggregated findings across all four third‐wave therapies and found some significant effects on psychological outcomes, wellbeing, functioning and pain. However, the results were limited by heterogeneity in both the therapeutic modalities and the sample characteristics, which complicates the attribution of outcomes to any single therapy. This review's broad scope, including acceptance and commitment therapy, CFT, mindfulness‐based cognitive therapy and metacognitive therapy, alongside diverse participant settings (schools to clinical service settings), makes it challenging to draw specific conclusions. Perkins et al. (2023) also reported that 34 of the 50 included studies used group interventions, again highlighting group delivery as the most common format for both children and adults.

The current evidence base suggests that groups are a common form of compassion‐focused intervention delivery (Millard et al. 2023; Craig et al. 2020; Perkins et al. 2023). The widespread use of groups is likely due, in part, to cost‐effectiveness, which funding bodies and commissioning groups consider when allocating healthcare resources (Craig et al. 2020; Perkins et al. 2023). Literature has also suggested that CFT is particularly well suited to a group format. For instance, a recent qualitative meta‐synthesis by Garrett et al. (2025) found that ‘the repeated, reciprocal sharing of experiences between group members and having these received by other group members with recognition, understanding, and compassion built a deep sense of connection and belonging for participants within the group’ (p. 18).

Overall, whilst a broader review of third‐wave therapies (Perkins et al. 2023) exists for this population, it included only two CFT studies and did not report their specific results. There is currently no review of the evidence base for compassion‐focused groups used with children and adolescents. This represents a notable gap in literature considering the increasing evidence base around the relationship between compassion and mental health difficulties in children and adolescents (Pullmer et al. 2019; Muris et al. 2024; Neuenschwander and von Gunten 2024).

### 1.5 Aims and Review Questions

A review of the evidence base for compassion‐focused groups for this population has yet to be conducted. Therefore, this review aims to answer the following research questions:

1. How have compassion‐focused group interventions been delivered to child and adolescent clinical populations within existing studies?
2. What are the compassion‐focused and wider psychological outcomes of these group interventions for clinical child and adolescent populations?
3. How methodologically robust are these studies?

## 2 Method

### 2.1 Protocol and Registration

This review was conducted in accordance with the Preferred Reporting Items for Systematic reviews (PRISMA; Page et al. 2021) guidelines, and the protocol was prospectively registered with the PROSPERO International Prospective Register of Systematic Reviews (reference: CRD42024524151).

### 2.2 Eligibility Criteria

The ‘Population, Intervention, Comparison, Outcome, and Study Design’ (PICOS) framework was used to guide the development of the eligibility criteria, to support efficient screening of studies (Methley et al. 2014; Amir‐Behghadami and Janati 2020). The inclusion and exclusion criteria have been detailed in Table 1.

**TABLE 1:** Eligibility criteria using the PICOS framework.

PICOS	Inclusion	Exclusion
Population	Children and adolescents: Aged 0–18 years. If sample included individuals 19 years and older, only included if the children's data presented separately. Scoring above a clinical cut‐off point on a relevant screening measure (e.g., PHQ‐A, GAD‐10, RCADS) AND/OR have a diagnosed physical health or mental health condition(s) AND/OR recruited from a clinical setting, including, but not limited to, mental health services (e.g., primary and secondary care, specialist services), learning disability services, physical health settings (e.g., paediatric health setting), looked after children services, and forensic settings	Non‐clinical population e.g., general population and universal interventions to school children Adults, above the age of 18 Parents of children with physical/mental health condition
Intervention	Studies must: Be primarily based upon CFT Be a group intervention Studies which use other therapeutic approaches alongside CFT, such as Mindful Self‐Compassion and Cognitively Based Compassion, will also be included if the primary intervention is CFT.	No distinct CFT component (e.g., studies exclusively using mindfulness‐based interventions will be excluded)
Comparison	The review will also include non‐controlled/comparison studies. If there is a comparator, studies that compare CFT to: Another psychological intervention, e.g., CBT or ACT Treatment as usual A control group A wait list control	
Outcome	Studies must include at least one outcome measure pre‐ and post‐intervention, such as: A compassion measure e.g., self‐compassion Measure a psychological construct such as shame, self‐criticism, psychological flexibility Measure mental health symptomology, such as, anxiety or depression Measure wellbeing and functioning, such as quality of life and adjustment	No compassion/psychological/or mental health outcome measures used
Study design	Quantitative studies only. These may include: Randomised controlled trials Non‐controlled studies Feasibility studies Pilot studies Multiple baseline design If a mixed methods design, only the quantitative elements will be extracted	Qualitative studies

### 2.3 Search Strategy

Relevant studies were identified by searching the following databases: MEDLINE/PubMed (Ovid); APA PsycINFO (Ovid); Embase (Ovid); Scopus; CINAHL/ERIC (EBSCO) in August 2024. The grey literature was also searched via the ProQuest Dissertation and Theses database. The decision to include grey literature was made to capture the emerging evidence base, and to reduce publication bias where studies that only report positive findings are published (Paez 2017). Please see Table 2, which displays the search terms used.

**TABLE 2:** Databases and search terms.

Title of database	Syntax (search terms) used
MEDLINE/PubMed (Ovid)	(TITLE‐ABS‐KEY ((compassion OR compassionate) W/5 (group* OR programme OR program OR intervention* OR training)) AND TITLE‐ABS‐KEY (child* OR teen* OR youth OR paediatric* OR pediatric* OR ‘young people’ OR ‘young person*’ OR adolescen*)) AND PUBYEAR > 2004 AND PUBYEAR < 2025 AND (LIMIT‐TO (LANGUAGE, ‘English’))
APA PsycINFO (Ovid)	
Embase (Ovid)	
Scopus	
CINAHL/ERIC (EBSCO)	((compassion OR compassionate) n5 (group* OR programme OR program OR intervention* OR training)) AND ((child* OR teen* OR youth OR paediatric* OR pediatric* OR ‘young people’ OR ‘young person*’ OR adolescen*))
ProQuest	noft((compassion OR compassionate) N/5 (group* OR programme OR program OR intervention* OR training)) AND noft((child* OR teen* OR youth OR paediatric* OR pediatric* OR ‘young people’ OR ‘young person’ OR ‘young persons’ OR adolescen*))

The search was limited to literature from 2005 onwards, reflecting the development of CFT in the early 2000s. Following the main databases search, an additional search was conducted on Google Scholar using terms such as, ‘compassion focused therapy children’ and ‘compassion focused therapy adolescents’ to identify additional studies. Retrieved papers were title and abstract screened against the eligibility criteria, followed by full‐text screening for those remaining. An ancestry and citation search were also performed with eligible full‐text articles, but no further studies meeting the inclusion criteria were identified. To ensure inter‐rater reliability, a second reviewer screened 25% of the 1090 titles/abstracts (*n* = 273), with three conflicts, yielding 98.9% agreement and a Cohen's kappa (κ) of 0.94. The same reviewer screened 25% of 108 full texts (*n* = 27), with one conflict, resulting in 96.3% agreement, and a kappa of 0.92. All conflicts were centred around whether the study sample could be deemed a clinical population. All conflicts were resolved through discussion until consensus was reached. EndNote and Rayyan were used to support the search.

### 2.4 Study Selection

**FIGURE 1:**
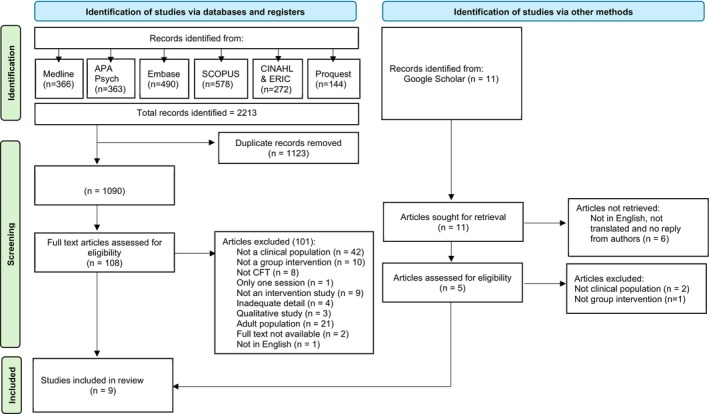
PRISMA flow diagram of search strategy.

The initial search identified 2213 papers. EndNote and Rayyan removed duplicates, reducing the studies to 1090. After the title and abstract screening, 108 of these met the inclusion criteria. After full‐text screening of these 108 papers, seven papers were deemed eligible to be included in the review. Eleven papers were screened off Google Scholar, and two were eligible for inclusion. A total of nine studies were included in the narrative synthesis. Please see Figure 1 for a PRISMA flow diagram overview of the study selection process.

### 2.5 Data Extraction

The following data was extracted: author, publication date; study location; number of participants; participant demographics (e.g., sex/gender, ethnicity, age); type of clinical sample; recruitment setting; study design; content of intervention; reference(s) that intervention was based upon; length of intervention; outcome measures used; data time points; and primary outcomes.

A narrative synthesis was conducted as there was considerable heterogeneity between studies. This review followed ‘The Synthesis Without Meta‐analysis’ (SWiM) guidelines (Campbell et al. 2020) which provides nine steps to transparently report on how studies are grouped, the standardised metric used for the synthesis, the synthesis method, presentation of data, synthesis findings and limitations (Campbell et al. 2020).

### 2.6 Quality Appraisal

The Psychotherapy Outcome Study Methodology Rating Form (POMRF; Öst 2008) was used to critically appraise the studies (see Supplementary File S2 and Supplementary File S3). The POMRF is a 22‐item scale which rates the methodological quality of varying research designs (i.e., from case series to controlled studies). It assesses various areas, including sample characteristics; study design; therapist training; psychometric properties of outcome measures; statistical analysis; bias in reporting of results. Each item is rated on a three‐point scale, ranging from poor (0), fair (1) to good (2). The total score is the sum of each of the items, ranging from 0 to 44. A higher score indicates greater methodological rigour. The POMRF was chosen due to its specific consideration to psychological therapy, such as, therapist competency, adherence to the model, and therapeutic confounding variables, e.g., medication. Additionally, it has been used in published reviews investigating third‐wave psychological therapies with children (Swain et al. 2015; Hulgaard et al. 2019; Harris and Samuel 2020). It has good internal consistency (Cronbach's alpha = 0.86) and inter‐rater reliability within the range of 0.50–1.00 (Öst 2008).

The POMRF was adapted for use within this current review. As most studies did not require a mental or physical health diagnosis, two items of the POMRF were excluded: item two (severity and chronicity of the disorder) and item four (reliability of the diagnosis in question). Therefore, the total POMRF scores ranged from 0 to 40. Each study was critically appraised by the author, and a second rater reviewed 50% of the studies. The two raters compared their scores, and where there were discrepancies, these were discussed until consensus was reached.

## 3 Results

The included studies involved a total of 171 children and adolescents, aged 11–17 years, recruited across six countries: Iran, Sweden, India, New Zealand, United Kingdom and United States. All studies were recent, with the three earliest being published in 2020 (Bratt et al. 2020; Boggiss et al. 2020; Joseph and Bance 2020). Eight studies were published journal articles, and one a master's dissertation (Tait 2022). Table 3 provides an overview.

**TABLE 3:** Overview of included studies.

Author(s) (location)	Design	Clinical group (recruited from)	*N*	Demographics	Intervention (reference)	Length of group	Outcome measures	
							Compassion	Other
Khosravi et al. (2022) (Iran)	Quasi experimental (pre–post with control group)	Body image disorder (psychological clinics)	15	Gender: 100% female Age: M = 13.26 Ethnicity: not recorded	CFT (Gilbert 2009)	10 weekly 90‐min sessions	Self‐Compassion Scale (SCS) The Levels of Self‐criticism (LOSC) Scale	The multidimensional Body‐Self Relations Questionnaire (MBSRQ)
Bratt et al. (2020) (Sweden)	Quasi experimental (pre‐post with control group)	Complex mental health (psychiatric outpatient clinic)	19	Gender: 83.7% female Age: 14–17 Ethnicity: not recorded	CFT (Gilbert 2009, 2010a)	8 sessions	Self‐Compassion Scale–Short Form (SCS‐SF)	Perceived Stress Scale (PSS)
Louis and Reyes (2023) (India)	Uncontrolled pre‐post (no control group)	Parental domestic violence and low self‐esteem (shelter homes for sexual abuse victims)	10	Gender: not recorded Age: 11–17 Ethnicity: not recorded	Cognitive self‐compassion (CSC)	8 modules 6 weeks	None	Coopersmith Self‐Esteem Inventory‐School form
Boggiss et al. (2020) (New Zealand)	Randomised waitlist‐controlled feasibility trial	Type 1 diabetes and disordered eating using screening tools (diabetes clinic)	11	Gender: 64% female Age: 12–16 Ethnicity: 55% NZ European	CFT Gilbert (2005) Germer and Neff (2019)	2 (2.5 h) sessions delivered 1 week apart	SCS‐SF	Diabetes Eating Problem Survey Revised (DEPS‐R) Self‐Care Inventory Problem Areas in Diabetes survey PSS
Damavandian et al. (2022) (Iran)	Uncontrolled pre‐post‐follow‐up (no control group)	History of self‐harm (Corrections and Rehabilitation Center)	13	Gender: not recorded Age: not recorded Ethnicity: not recorded	CFT (Gilbert 2010a)	12 weeks (1, 90‐min session per week)	None	Inventory OF Statements About Self‐Injury (ISAS) The Emotional Self‐Regulation Questionnaire The aggression questionnaire
Lau‐Zhu and Vella (2023) (England)	Uncontrolled pre‐post (no control group)	Care system (specialist psychology team)	8	Gender: 100% female Age: 12–16 Ethnicity: 75% white, 25% black	CFT Gilbert (2014)	8 weeks (1, 90‐min session per week)	Visual analogue scale ‘I am kind to myself about my struggles’	RCADS‐47 (parent version) RCADS‐11 (child version) Child version of the Outcome Rating Scale
Tait (2022) (England)	Mixed method Uncontrolled pre‐post (no control group)	Severe mental health difficulties	10	Gender: 5 Cis Male, 3 Cis Female, 2 Gender minority Age: 13–17 Ethnicity: 80% white, 20% mixed race	CFT Gilbert (2014)	13 weekly 1–1.5‐h Zoom sessions	SCS‐Y	Behaviour and feelings survey Youth (BFSY) Depression, anxiety & stress scales‐ short form (DASS‐21)
Bluth et al. (2024) (USA)	Uncontrolled pre‐post (no control group)	Suicidal ideation and mental health concerns Depression threshold measure (gender clinics)	34	Gender: 52% transmasculine Age: 13–17 Ethnicity: 77% white	Mindful self‐compassion (MSC)	8 sessions (twice weekly over 4 weeks)	SCS‐Y	Pediatric Depression Scale (PROMIS) Connor‐Davidson Resilience Scale (CD‐RISC) Interpersonal Needs Questionnaire (INQ) Gender Minority Stress and Resilience Measure (GMSRM) Depression Symptom Index–Suicidality Subscale (DSI‐SS)
Joseph and Bance (2020) (India)	RCT	Trauma, low self‐compassion and high shame (shelter homes for sexual abuse victims)	18	Gender: 100% female Age: 12–17 Ethnicity: not recorded	Compassion‐focused Visual Art Therapy (CVAT)	14 sessions (twice weekly)	SCS	Trauma‐Related Shame Inventory (TRSI)

*Note:* N reflects the number of participants who received the compassion‐focused group intervention, not the total study sample size. Wider demographics reflect the total study sample size. Age is reported as a range (minimum–maximum). For Khosravi et al. (2022), age is reported as mean (M) because the range was not provided. Uncontrolled pre‐post = one‐group design without a control group; Quasi‐experimental = non‐randomised comparison between intervention and control groups.

### 3.1 Methodological Quality

Study quality was assessed using the POMRF (Öst 2008), with scores and subsequent rating shown in Table 4. Scores ranged from 8 to 23, out of 40 (M = 14.7, SD = 5.28). As Öst (2008) has not provided a rating range, this review developed one following the procedure outlined by Swain et al. (2013) and Knight and Samuel (2022). The ‘below average’ range (0–9) was calculated as one standard deviation below the mean (9). The ‘average’ range (10–20) was calculated as within one standard deviation of the mean (20). The ‘above average’ range (21+) was calculated as one standard deviation above the mean. This was developed to enable comparison of the methodological quality between the included studies. Most studies fell within the ‘average’ range (*n* = 5, 55.6%), with two studies rated as ‘below average’, and two as ‘above average’.

**TABLE 4:** POMRF score and rating.

Study	POMRF score	Rating
Louis and Reyes (2023)	8	Below average
Damavandian et al. (2022)	8	Below average
Tait (2022)	13	Average
Khosravi et al. (2022)	13	Average
Lau‐Zhu and Vella (2023)	14	Average
Boggiss et al. (2020)	15	Average
Joseph and Bance (2020)	17	Average
Bratt et al. (2020)	22	Above average
Bluth et al. (2024)	23	Above average

The following sections expand on the methodological strengths and weaknesses of the included studies, organised according to themes of the POMRF items. Please see Supplementary File S1 for an extensive narrative synthesis.

#### 3.1.1 Clarity and Representativeness of Sample

In terms of sex of participants, five studies recorded sex as binary categorisation (male and female). In all five, participants were the majority female, with three samples being all‐female. Two studies were more inclusive, recognising gender diversity by allowing participants to identify beyond male and female categories (Tait 2022; Bluth et al. 2024). Two studies did not record the sex or gender of participants (Damavandian et al. 2022; Louis and Reyes 2023).

Regarding participant ethnicity, five studies did not record this (Khosravi et al. 2022; Bratt et al. 2020; Louis and Reyes 2023; Damavandian et al. 2022; Joseph and Bance 2020). Three of the four studies that recorded ethnicity reported a majority white sample (Bluth et al. 2024; Tait 2022; Lau‐Zhu and Vella 2023).

There was high heterogeneity in the types of populations recruited, and how they were screened to be included as a clinical sample. There were two studies which required a diagnosed mental health disorder; one a body image disorder (Khosravi et al. 2022) and the other no specific disorder requirement (Bratt et al. 2020). Additionally, there was one study which required a diagnosed physical health condition (type 1 diabetes; Boggiss et al. 2020). There were three studies which included children and young people who have experienced trauma (Lau‐Zhu and Vella 2023; Louis and Reyes 2023; Joseph and Bance 2020). Screening measures were used by four of the studies, which included low self‐esteem (Louis and Reyes 2023), low self‐compassion and high trauma‐related shame (Joseph and Bance 2020), disordered eating (Boggiss et al. 2020), and anxiety and depression (Tait 2022). Other more informal approaches were taken, which included screening for self‐harm (Damavandian et al. 2022) and attempted suicide or suicidal ideation (Bluth et al. 2024). Lau‐Zhu and Vella (2023) did not report a specific inclusion criterion but reflected on their sample's experiences of trauma, neurodiversity, low mood and self‐harm. These clinical samples were recruited from multiple settings: psychology services (2), psychiatric outpatient (1), protective housing facility (shelter home) (2), paediatric clinic (1), forensic setting (1), gender clinic (1) and an unknown setting (1). Tait (2022) did not provide details of their recruitment setting but used a screening threshold measure of anxiety/depression to determine a clinical sample.

#### 3.1.2 Intervention Format and Delivery

The duration of the intervention was reported by all studies, although there was large variability in length. The shortest intervention was Boggiss et al. (2020) who held two 2.5‐h sessions, 1 week apart. The longest intervention duration was Tait (2022) whose study delivered 13 weekly 1–1.5‐h sessions.

In terms of therapist training and competence, there were four studies which did not record what therapist(s) delivered the intervention (Louis and Reyes 2023; Damavandian et al. 2022; Tait 2022; Joseph and Bance 2020). Checks for adherence to the protocol and therapist competence were extremely limited across the studies. Six studies scored poorly on both treatment adherence and therapist competence (Tait 2022; Lau‐Zhu and Vella 2023; Damavandian et al. 2022; Boggiss et al. 2020; Bratt et al. 2020; Khosravi et al. 2022). Bluth et al. (2024) was the only study to score good for treatment adherence by using weekly checklists completed by a research assistant who sat in on the groups.

#### 3.1.3 Study Design

Eight of the nine included studies were scored as poor on the POMRF for study design. Four studies were the lowest quality, due to using a within‐groups design with no control group (Lau‐Zhu and Vella 2023; Bluth et al. 2024; Tait 2022; Louis and Reyes 2023). Additionally, Damavandian et al. (2022) described their study as quasi‐experimental, although it was not clear whether they used randomization and there were no details or outcomes of a control group. Therefore, going forward, this study was considered to not have compared the intervention to a control group.

Four studies compared an experimental and control group (Khosravi et al. 2022; Bratt et al. 2020; Boggiss et al. 2020; Joseph and Bance 2020). Two studies used treatment as usual (TAU; Bratt et al. 2020; Khosravi et al. 2022). Bratt et al. (2020)’s TAU included cognitive behavioural therapy, systematic psychological treatments, clinical assessments and psychosocial support. Khosravi et al. (2022) TAU utilised psychology therapies as before, but did not specify which. One study had a control group which received no treatment (Joseph and Bance 2020). The remaining study by Boggiss et al. (2020) had a waitlist control group and reported the challenge of being a fully powered trial due to their limited sample size. The outcomes of the studies will be interpreted in the context of the methodological quality of each studies design.

#### 3.1.4 Statistical Data

There were only two studies which used power calculations (Bratt et al. 2020; Damavandian et al. 2022). Only three studies used a follow‐up data point: Damavandian et al. (2022) had a 1‐month follow‐up, Bluth et al. (2024) used a 2‐month follow‐up, whilst Bratt et al. (2020) had the longest follow‐up, which was 6 months post‐group. However, the response rate was low, and they only reported on pre–post data. Four studies did not report effect sizes of their results (Louis and Reyes 2023; Boggiss et al. 2020; Lau‐Zhu and Vella 2023; Joseph and Bance 2020). Thus, making it difficult to interpret the magnitude of the differences found.

#### 3.1.5 Outcome Measures

##### 3.1.5.1 Compassion‐Focused Measures

Out of the nine included studies, seven used a specific compassion‐focused outcome measure, whilst two studies did not (Louis and Reyes 2023; Damavandian et al. 2022). The main measure used was the Self‐Compassion Scale (SCS) developed by K. D. Neff (2003b) and comes in three formats: the full SCS, a short form (SCS‐SF) and an adapted version for youth (SCS‐Y). These are reliable and valid measures (K. D. Neff 2016; Raes et al. 2011; K. D. Neff et al. 2021) which have been developed for specific age ranges. The SCS was developed for ages 14 and above, and the SCS‐Y is designed for early adolescents aged 10–14. There were two studies that used the SCS, and both included participants younger than 14 years. Khosravi et al. (2022) used this measure despite the mean age of participants being 13, and Joseph and Bance (2020) also used this whilst having participants ranging from 12 to 17 years of age. Of the two studies that used the SCS‐SF, Bratt et al. (2020) used this measure with an appropriate sample age range of 14–17, whilst Boggiss et al. (2020) used this despite having participants whose ages ranged from 12 to 16. The SCS‐Y was used by two studies: Tait (2022) and Bluth et al. (2024) who both used this regardless of some of their samples being older than age 14 (range of 13–17).

There were two additional measures used. Lau‐Zhu and Vella (2023) developed their own single visual analogue scale stating, ‘I am kind to myself about my struggles*’* which had a rating scale of ‘completely disagree’ to ‘completely agree’. Khosravi et al. (2022) used the Levels of Self‐criticism Scale (Thompson and Zuroff 2004), however, this measure has not been validated for use with children.

##### 3.1.5.2 Other Measures

Many other outcome measures were used inconsistently to measure mental health domains and psychological constructs. Two studies (Bratt et al. 2020; Boggiss et al. 2020) used the Perceived Stress Scale (PSS; Cohen et al. 1983). Three studies had a common theme of measuring anxiety and/or depression (Lau‐Zhu and Vella 2023; Tait 2022; Bluth et al. 2024). There were different tools used to measure this—the Revised Child Anxiety and Depression Scale (Chorpita et al. 2015), Depression, Anxiety and Stress Scale (DASS‐21; Henry and Crawford 2005), Pediatric Depression Scale (PROMIS; Kaat et al. 2020) and Depression Symptom Index–Suicidality Subscale (DSI‐SS; Metalsky and Joiner 1997).

The reliability and validity of outcome measures used was variable across studies. Five measures used have not been validated for use with children and adolescents. The DASS (Henry and Crawford 2005) has three constructs (depression, anxiety and stress) and has been found to not adequately differentiate between these in adolescent populations (Moore et al. 2017). The other four unvalidated measures were as follows: Trauma‐Related Shame Inventory (Øktedalen et al. 2014), Inventory of Statements About Self‐Injury (Klonsky and Glenn 2009), The Emotional Self‐Regulation Questionnaire (Hofmann and Kashdan 2010), The Aggression Questionnaire (Buss and Perry 1992). Moreover, there were two measures used which have been validated for this population but were designed for a specific age range which the study sample did not fall within. The multidimensional Body‐Self Relations Questionnaire (MBSRQ; Cash 2000) was devised for adolescents aged 15 and above, but was used by Khosravi et al. (2022) whose sample had a mean age of 13. Additionally, the DSI‐SS (Metalsky and Joiner 1997) has been validated for ages 15 and above, but was used by Bluth et al. (2024) whose sample included participants from age 13. Bratt et al. (2020) was the only study to score good on the POMRF as all of their outcome measures had good psychometric properties and were the best available to the authors.

### 3.2 Study Outcomes

Table 5 presents the mixed compassion and mental health outcomes. The table is organised by outcome variable, with studies within each category ordered by methodological quality (highest to lowest). There were three measures of effect size utilised. The most frequently used was Cohen's *d* which has been interpreted using Cohen's (1988) criteria: small effect (*d* = 0.2), medium effect (*d* = 0.5), large effect (*d* = 0.8 or greater). For studies using Hedges *g*, the criteria were: small effect (*g* = 0.2), medium effect (*g* = 0.5) and a large effect (*g* = 0.8). The third measure of effect used was partial eta squared. Cohen's (1988) guidance was again used to interpret a small effect (n2 = 01), a medium effect (n2 = 0.06) and a large effect (n2 = 0.14 or greater).

**TABLE 5:** Study intervention outcomes.

Outcome variable	Study (conditions)	Main outcome (time points)
		P (effect size)
**Self‐compassion**	Bluth et al. (2024) (MSC only)	Compassion increased (pre‐follow‐up) Not recorded (moderate‐large ES—*d* = 0.7)
	Bratt et al. (2020) (CFT vs. TAU)	No significant difference in self‐compassion between CFT and TAU groups (pre–post) 0.34 (very small ES—*g* = 0.10)
	Joseph and Bance (2020) (CFT vs. NTG)	Higher levels of self‐compassion (pre–post) 0.05 (ES not recorded)
	Boggiss et al. (2020) (CFT vs. waitlist)	Mean changes in compassion were relatively small (pre–post) Not recorded (ES not recorded)
	Khosravi et al. (2022) (CFT vs. TAU)	CFT significantly improved self‐compassion rates (pre–post) >0.05 (large ES—η2 = 0.345)
	Tait (2022) (CFT only)	No significant differences in compassion ratings (pre–post) 0.28 (small ES—*g* = 0.37)
**Self‐criticism**	Khosravi et al. (2022) (CFT vs. TAU)	CFT significantly reduced self‐criticism rates (pre–post) >0.05 (large ES—η2 = 0.363)
**Stress**	Bratt et al. (2020) (CFT vs. TAU)	No significant difference in stress ratings between CFT and TAU groups (pre–post) 0.34 (small ES—*g* = 0.14)
	Boggiss et al. (2020) (CFT vs. waitlist)	Mean changes in stress were relatively small (pre–post) Not recorded (ES not recorded)
**Anxiety and depression**	Bluth et al. (2024) (MSC only)	Depression symptoms decreased (pre‐follow‐up) Not recorded (small ES—*d* = − 0.19)
	Lau‐Zhu and Vella (2023) (CFT only)	Five of the sample's parents reported improvements in their child's anxiety and low mood. Two children reported reductions (pre–post) Not recorded (ES not recorded)
	Tait (2022) (CFT only)	No significant differences in anxiety/depression ratings (pre–post) 0.21 (small‐medium ES—*g* = −0.44)
**Self‐harm**	Damavandian et al. (2022) (CFT only)	Significant effect in reducing self‐harm behaviours (pre–post) 0.05 (large ES—n2 = 0.918)
**Suicidality**	Bluth et al. (2024) (MSC only)	Suicidality decreased (pre–post) Not recorded (large ES—*d* = −0.99)
**Self‐esteem**	Louis and Reyes (2023) (CSC only)	Significant difference between scores in self‐esteem (pre–post) 0.05 (ES not recorded)
**Internalising and externalising problems**	Tait (2022) (CFT only)	No significant differences for reported problems (pre–post) 0.7 (small ES—*g* = 0.13)
**Trauma‐related shame**	Joseph and Bance (2020) (CFT vs. NTG)	Lower levels of trauma‐related shame compared to the NTG (pre–post) 0.05 (ES not recorded)
**Diabetes outcomes: disordered eating; diabetes distress; self‐care**	Boggiss et al. (2020) (CFT vs. waitlist)	Mean changes were relatively small across all outcomes (pre–post) Not recorded (ES not recorded)
**Emotion regulation**	Damavandian et al. (2022) (CFT only)	Effective for two components of emotion regulation (adaptive and tolerance), not concealing (pre–post) 0.05 (adjusting = large ES—n2 = 0.312) (concealing = small ES—n2 = 0.091) (tolerating = large ES—n2 = 0.839)
**Aggression** **Anger** **Hostility**	Damavandian et al. (2022) (CFT only)	Significant effect in reducing aggression, anger and hostility (pre‐follow‐up) 0.05 (physical aggression = large ES—n2 = 0.715) (verbal aggression = large ES—n2 = 0.732) (anger = large ES—n2 = 0.822) (hostility = large ES—n2 = 0.748)
**Interpersonal needs**	Bluth et al. (2024) (MSC only)	Burdensomeness and belonging went down, (pre‐follow‐up) Not recorded (burdensomeness = small ES—*d* = −0.29) (belonging = small ES—*d* = −0.16)

Abbreviations: CG, control group; ES, effect size; NTG, no treatment.

#### 3.2.1 Self‐Compassion

Self‐compassion was measured in seven studies with mixed findings that varied according to study design and comparator quality.

##### 3.2.1.1 Controlled Designs

Three studies employed comparator conditions. Bratt et al. (2020) using a TAU control found no significant differences between CFT and TAU, with a very small effect size (*g* = 0.10). Similarly, Boggiss et al. (2020) utilised a waitlist control group and reported relatively small mean changes in compassion following intervention; however, effect sizes were not reported. In contrast, Khosravi et al. (2022) found a statistically significant increase in self‐compassion with a large effect size (η2 = 0.345).

##### 3.2.1.2 No‐Treatment Control Designs

One study compared CFT with no treatment. Joseph and Bance (2020) reported higher levels of self‐compassion post‐group but did not report on effect size.

##### 3.2.1.3 Uncontrolled Designs

Three studies did not compare the intervention to a control group. Bluth et al. (2024) found compassion increased at follow‐up with a moderate‐large effect size (*d* = 0.7). Lau‐Zhu and Vella (2023) found that only two of their participants perceived an increase in self‐compassion post‐group, and Tait (2022) found no significant differences in compassion ratings (*g* = 0.37).

#### 3.2.2 Self‐Criticism

A TAU controlled study by Khosravi et al. (2022) measured levels of self‐criticism alongside self‐compassion and found a statistically significant reduction in self‐criticism following CFT. Therefore, engaging with CFT was associated with lower levels of self‐criticism compared to receiving no treatment. This result had a large effect size (n2 = 0.363), indicating that group membership accounted for 36.3% of the variance in self‐criticism scores.

#### 3.2.3 Stress

Two controlled studies used the PSS to measure the impact of a CFT group on stress. Bratt et al. (2020) using a TAU control, found no significant difference in stress ratings between the CFT and treatment as usual group (*g* = 0.14). Similarly, Boggiss et al. (2020) found relatively small changes in stress levels following the CFT group, compared to a waitlist control.

#### 3.2.4 Anxiety and Depression

Three uncontrolled studies investigated the impact of a CFT group on anxiety and depression ratings. Bluth et al. (2024) found a reduction in depression ratings, but with a small effect size (*d* = − 0.19). Lau‐Zhu and Vella (2023) found that five of their sample's parents reported improvements in their child's anxiety and low mood, and two children reported reductions in anxiety and depression. However, due to the limited data and statistical analysis provided in this study, these results should be viewed cautiously. Tait (2022) found no significant differences in anxiety and depression ratings post‐group using the DASS (*g* = −0.44). As the DASS is not validated for children and young people and has been found to not differentiate well between its three substructures of depression, anxiety and stress (Moore et al. 2017), this may in part account for this finding.

#### 3.2.5 Other Outcomes

There were other outcomes which were measured infrequently between the studies. Each of the following domains was only measured by one individual study, and so comparison across the literature was not possible.

##### 3.2.5.1 Controlled Designs

Boggiss et al. (2020) conducted a paediatric study and explored diabetes specific outcomes (disordered eating, diabetes distress and self‐care). They found small changes across all domains but did not report on the effect sizes of these changes.

##### 3.2.5.2 No‐Treatment Control Designs

Trauma‐related shame was investigated by Joseph and Bance (2020) who found that when CFT was compared to no treatment, CFT led to lower levels of shame. The effect size was not recorded.

##### 3.2.5.3 Uncontrolled Designs

Bluth et al. (2024) found that suicidality of the participants decreased, and this had a large effect size (*d* = −0.99). They also investigated interpersonal needs within the transgender population and found that both burdensomeness and thwarted belongingness reduced at follow‐up. The effect sizes were small. Tait (2022) investigated the impact on internalising and externalising problems and found no significant differences pre‐ to post‐group. Damavandian et al. (2022) found that CFT led to a reduction in self‐harming behaviours, and this had a large effect size (n2 = 0.918). They also measured emotion regulation and found that CFT was effective and had large effect sizes for two components of this, adaptive and tolerance, but a small effect size for the element of concealing. This study also measured aggression, anger and hostility and found that CFT had a significant large effect on these three areas. Louis and Reyes (2023) found that self‐esteem significantly improved; however, they did not report on the effect size.

## 4 Discussion

One aim of the current review was to explore how compassion‐focused group interventions have been delivered to child and adolescent clinical populations. The nine included studies recruited young people aged between 11 and 17 years old. Most of the samples were reported as majority female participants. These samples were recruited from Iran, Sweden, India, New Zealand, United Kingdom and United States. There were varied clinical groups, including young people with a diagnosed mental health disorder (e.g., body image disorder), a physical health condition (e.g., diabetes) and trauma histories. Other subgroups included young people experiencing low self‐esteem, low self‐compassion, high trauma‐related shame, disordered eating, anxiety/depression and suicidality or engaging in self‐harm. These samples were recruited from varied settings including psychology and psychiatric services, protective housing facilities, a paediatric clinic, a forensic setting and a gender service. The mean number of group sessions delivered was 9.7, but the range of sessions across studies was 2–14.

Another aim of this review was to review the compassion and psychological outcomes of these interventions. No studies had an active control group which compared CFT to an alternative therapy. Only two studies examining compassion outcomes utilised comparator control groups. Bratt et al. (2020) compared CFT to TAU and found no significant between‐group differences, with a very small effect size (*g* = 0.10). Similarly, Boggiss et al. (2020) compared the intervention with a waitlist control and reported small mean differences in compassion, although the effect sizes were not reported.

Two studies compared the intervention with no‐treatment controls. Both reported increased self‐compassion outcomes following the group intervention (Joseph and Bance 2020; Khosravi et al. 2022). Joseph and Bance (2020) did not report effect size and Khosravi et al. (2022) found a large effect (η2 = 0.345).

The three uncontrolled studies reported mixed compassion outcomes. Bluth et al. (2024) found increased self‐compassion at follow‐up with a moderate‐large effect size (*d* = 0.7). In contrast, Lau‐Zhu and Vella (2023) reported only two participants perceived an increase in self‐compassion post‐group (no effect size reported). Tait (2022) found no significant differences in compassion ratings (*g* = 0.37).

This review also synthesised wider mental health and wellbeing outcomes and the outcomes were similarly mixed. Among the three studies that compared the intervention to a control, Bratt et al. (2020) found no difference between the intervention and TAU for ratings of stress (*g* = 0.14), while Boggiss et al. (2020) reported a small change in stress ratings following intervention, compared to a waitlist control. Boggiss et al. (2020) also investigated diabetes‐specific outcomes, including disordered eating, diabetes distress and self‐care, reporting small changes across all domains, although effect sizes were not provided. Khosravi et al. (2022) reported a statistically significant reduction in self‐criticism, with a large effect (n2 = 0.363).

One study compared the group intervention with a no‐treatment control. Joseph and Bance (2020) found lower levels of trauma‐related shame compared to no treatment (no effect size reported).

Five uncontrolled studies investigated broader mental health outcomes. Three examined anxiety and depression. Bluth et al. (2024) reported reductions in depression ratings with a small effect (*d* = −0.19). Lau‐Zhu and Vella (2023) reported that participants perceived changes in anxiety and mood, though no effect sizes were recorded. Tait (2022) found no difference in anxiety or depression (*g* = −0.44). Other outcomes reported in uncontrolled studies included reductions in self‐harming (Damavandian et al. 2022; n2 = 0.918) and improvements in self‐esteem with no effect size reported (Louis and Reyes 2023).

### 4.1 Interpretation of Findings

Consideration of the overall plausibility and credibility of the evidence base highlights several important considerations. Studies were typically characterised by small sample sizes, which limited statistical power. Larger effect sizes and more favourable outcomes were predominantly reported in uncontrolled or no‐treatment comparison designs. This raises the likelihood that improvements reflect natural change or other confounding variables, rather than intervention‐specific effects. In contrast, studies which utilised a TAU or waitlist control often reported smaller or non‐significant effects. Therefore, the impact of compassion‐focused interventions for children and adolescents may be more modest than indicated by the studies with weaker designs. Additionally, inconsistency in reporting of effect sizes further limits interpretation and confidence in the results of some studies. Future research would benefit from larger, more highly powered trials to develop this emerging field.

The varied complexity in presentations across the samples is notable and may explain the differing outcomes. Bratt et al. (2020) recruited young people who were under psychiatric care at a child and adolescent psychiatric outpatient clinic, many of whom had a diagnosed mental health disorder. In comparison, Bluth et al. (2024) recruited a community sample of young people who identified as transgender and met the criteria for low mood and suicidality. Given the more general nature of the population in Bluth et al. (2024)'s study, participants may have experienced fewer emotional and cognitive barriers to engaging with a CFT group intervention.

Psychological constructs, including shame, self‐criticism and compassion, are inherently complex, requiring introspection and abstract thinking. This may be challenging for adolescents, whose cognitive and emotional development is still maturing (Gilbert and Irons 2009; Moshman 2020). This issue was highlighted by Bratt et al. (2020) who presented several potential reasons for their lack of significant findings, including adolescents' difficulty engaging with the abstract concepts of mentalising and CFT. This may be due to ongoing development of formal operational thinking, as outlined in Piaget's theory of cognitive development (Piaget 1952; Piaget 1972). Participants in this review were aged 11–17, and Piaget's theory suggests that whilst adolescents begin to develop metacognition and abstract thinking, the ability to do so might not be consistent across contexts. Additional emotional factors such as bullying, peer rejection and difficulties within their attachment relationships may further hinder adolescents' ability to mentalise and regulate emotions, which could limit their engagement with CFT (Gilbert and Irons 2009). As such, the cognitive demands of CFT might exceed the developmental stage of adolescents unless appropriately adapted.

Differences in how CFT was delivered might also explain the mixed compassion findings. Of the three studies which reported significant improvements in compassion, two combined CFT with other therapeutic approaches—mindfulness (Bluth et al. 2024) and art therapy (Joseph and Bance 2020). Contrastingly, the four studies which did not find a significant improvement all used CFT as a standalone intervention. There is a possibility that integrating CFT with other approaches may enhance its effectiveness for children and adolescents. Such integration may support young people in engaging with abstract or emotionally complex CFT concepts. For instance, combining CFT with mindfulness practices, like Bluth et al. (2024)'s study, has been argued to foster a greater embodied experience of compassion, rather than emphasizing didactic cognitive learning (Gilbert and Simos 2022). However, combining CFT with other therapies makes it harder to isolate the effects of CFT alone.

Another possible factor is whether studies appropriately adapted CFT for this population. Carona et al. (2017) argued that CFT for younger populations should not only focus on the individual, but their specific needs within their interpersonal context, such as their home, school, culture, community, peer groups and parent–child relationships. Few studies in this review appeared to report on such adaptations, although Bratt et al. (2020) delivered a parallel parent group. The limited reporting of adaptations may partially explain the limited or mixed findings, as studies may not have addressed the systemic sources of threat which children often experience (Carona et al. 2017). Further research should explore whether standalone CFT or integrated approaches are more suitable and effective for this population, alongside whether adaptations that involve family and the young person's network enhance the outcomes of CFT.

### 4.2 Limitations of Included Studies

Another key limitation of the reviewed studies was their use of unvalidated outcome measures for child and adolescent populations. Five measures used have not been validated for this population. Outcome measures should be developmentally appropriate (Kwan and Rickwood 2015), as children may struggle to understand measures designed for adults (Thapa Bajgain et al. 2023). For example, Morey et al. (2024) adapted the adult CompACT measure for children and found that young people had challenges in comprehending all 23 adult items. It is essential to validate measures for this population to ensure they accurately assess intended outcomes (McNeill et al. 2021).

An additional limitation was the misalignment of outcome measures with intervention goals. For instance, Louis and Reyes (2023) delivered a cognitive self‐compassion intervention, but only measured self‐esteem, potentially overlooking changes in cognitions and self‐compassion. Similarly, despite the primary aim of CFT being to increase self‐compassion (Gilbert 2010b), two studies did not use any measure of compassion (Louis and Reyes 2023; Damavandian et al. 2022). It is possible their interventions were effective in this area, but this was not adequately assessed.

### 4.3 Limitations of Systematic Review

A key limitation of this review was the considerable heterogeneity of intervention designs and reporting of methodological limitations across the included studies. There was an overall lack of transparency in reporting therapist training, adherence to model and sample characteristics. Other limitations included small sample sizes and a lack of control groups. These limitations made it difficult to draw conclusions about the effectiveness of group interventions, alongside limiting the replicability of the studies. The methodological limitations identified in this review align with the wider evidence base. For instance, the review of third‐wave therapies by Perkins et al. (2023) observed similar methodological weaknesses.

Another limitation of this review is the use of the POMRF (Öst 2008) which lacks a rating scale to compare the quality of studies across reviews. A scale had to be devised using insights from previous reviews (Swain et al. 2013; Knight and Samuel 2022) which allowed the methodological quality of each study to be rated relative to the others, with most studies being assessed as ‘average’. As there are no other CFT systematic reviews which use the POMRF, there is currently no normative data with which to compare. This makes it challenging to determine whether the predominant ‘average’ ratings are meaningful. Until a standardised rating scale exists, these scores should be interpreted with caution.

Additionally, when using the POMRF, there is potential variation in how certain items are interpreted. For example, item eight (‘assessor training’) evaluates training in administering outcome measures. This item is biased towards studies which require professional training to administer psychometric measures, whilst many of the studies in this review used self‐report questionnaires. Therefore, caution is required when comparing POMRF scores, as any difference in how items are interpreted would limit the comparability of scores.

A further challenge was defining a ‘clinical’ population. Society has moved away from heavily stigmatising young people with a mental health diagnosis (Timimi 2015), however, as many studies did not report formal diagnoses, this complicated efforts to define a clinical group. Some studies used symptom thresholds on outcome measures to determine clinical and subsyndromal groups; however, these varied widely between studies. Therefore, the severity of participants' distress likely differed substantially across studies and may have contributed to the inconsistency in outcomes.

### 4.4 Implications

This review highlights the need for improved methodological standards in measuring outcomes for children and adolescents. Future studies should focus on validating outcome measures, so they are developmentally appropriate and reliable for young people, and to ensure clinically meaningful use (Kwan and Rickwood 2015). Moreover, compassion measures are not designed with clinical thresholds, making it difficult to determine whether changes in scores reflect clinically meaningful change. Future research would benefit from developing validated compassion measures with thresholds or consistently pairing these with measures that include clinical cut‐offs. This would strengthen conclusions regarding clinical change. Overall, there is a need for greater consistency in valid outcome measures used across studies, which would enable more rigorous comparison of outcomes and likely lead to higher quality research in this area (Black et al. 2023).

Another key implication is the need for a standardised rating scale for the POMRF (Öst 2008). Developing a scoring system with normative data would increase comparability between studies and improve the robustness of ratings within systematic reviews.

Finally, higher quality and more highly powered studies are required to strengthen the evidence base. RCT's would strengthen the reliability and validity of findings and guide best practice in this field. This would help to further establish the efficacy of CFT, identify optimal delivery formats (e.g., 1:1 or group‐based), and inform how CFT can be tailored to different settings and subpopulations. Given the methodological progress in adult CFT research (Millard et al. 2023), studies with younger populations may benefit from adopting the best practices from adult research.

## 5 Conclusion

In conclusion, this review examined an emerging area of clinical practice, with all included studies published from 2020 onwards. This review provides an early insight into the use of CFT groups for children and adolescents. The compassion and psychological outcomes were mixed, and the overall methodological quality of the studies was very limited. High heterogeneity in the study designs, the inconsistent use of outcome measures, and the challenges with defining a clinical population made it difficult to draw firm conclusions. Therefore, higher quality, more rigorously designed research is required to establish the efficacy of CFT groups for children and adolescents and inform future clinical practice in this area.

## Funding

This work was completed in partial fulfilment of a doctorate in clinical psychology funded by Health Education and Improvement Wales (HEIW).

## Conflicts of Interest

The authors declare no conflicts of interest.

## Supporting information


**Data S1:** Full narrative synthesis.
**Data S2:** Psychotherapy Outcome Study Methodology Rating Form (POMRF).
**Data S3:** POMRF ratings for included studies.

## Data Availability

All data extracted is included in the manuscript and supporting information.
