# Supplementary material for: The Use and Outcomes of Compassion‐Focused Group Interventions With Children and Adolescent Clinical Populations: A Systematic Review and Narrative Synthesis
Source: Clin Psychol Psychother. 2026 Mar 22;33(2):e70259. doi: 10.1002/cpp.70259 (PMC13006158; doi:10.1002/cpp.70259)
Supplement: Supplementary file 1 — Data S1: Full narrative synthesis. Data S2: Psychotherapy Outcome Study Methodology Rating Form (POMRF). Data S3: POMRF ratings for included studies. [file CPP-33-e70259-s001.docx]

**Supplementary File S1** - Full Narrative Synthesis

***Clarity and representativeness of sample***

In terms of sex of participants, five studies recorded sex as binary categorisation (male and female). In all five, participants were majority female, with three samples being all-female. There were two studies which were more inclusive, and recognised gender diversity by allowing participants to identify beyond male and female categories (Tait, 2022; Bluth et al. 2024). Tait (2022)’s sample included cis men (n=5), cis women (n=3) and gender minorities (n=2). Bluth et al. (2024) sample included transgender women (n=3), transgenders men (n=17), and nonbinary/gender queer/gender fluid (n=11) individuals. Out of the nine included studies, two did not record the sex or gender of participants (Damavandian et al., 2022; Louis & Reyes, 2023).

Regarding participant ethnicity, five studies did not record this (Khosravi et al., 2022; Bratt et al., 2020; Louis & Reyes, 2023; Damavandian et al., 2022; Joseph & Bance, 2020). Three of the four studies which recorded ethnicity reported a majority white sample (Bluth et al., 2024; Tait, 2022; Lau-Zhu & Vella, 2023) and Boggiss et al. (2022) a majority New Zealand European sample. Only one study, Joseph and Bance (2020), detailed the samples religion (58.3% Hindu).

There was high heterogeneity in the types of populations recruited, and how they were screened to be included as a clinical sample. There were two studies which required a child/adolescent to be diagnosed with a mental health disorder; one a body image disorder (Khosravi et al., 2022) and the other no requirement for a specific disorder (Bratt et al., 2020). Additionally, there was one study which required a diagnosis of a physical health condition (type 1 diabetes; Boggiss et al., 2020). There were three studies which included children and young people who have experienced trauma (Lau-Zhu & Vella, 2023; Louis & Reyes, 2023; Joseph & Bance 2020). Screening measures were used by four of the studies, which included low self-esteem (Louis & Reyes, 2023), low self-compassion and high trauma-related shame (Joseph & Bance, 2020), disordered eating (Boggiss et al., 2020), and anxiety and depression (Tait, 2022). Other more informal approaches were taken, which included screening for self-harm (Damavandian et al.,2022) and attempted suicide or suicidal ideation (Bluth et al., 2024). Lau-Zhu and Vella (2023) did not report a specific inclusion criterion but reflected on their sample’s experiences of trauma, neurodiversity, low mood, and self-harm. These clinical samples were recruited from multiple settings: psychology services (2), psychiatric outpatient (1), protective housing facility (shelter home) (2), paediatric clinic (1), forensic setting (1), gender clinic (1), and an unknown setting (1). Tait (2022) did not provide details of their recruitment setting but used a screening threshold measure of anxiety/depression to determine a clinical sample.

The most common reasons for participants being excluded were if a child or adolescent experienced - psychotic symptoms (2), substance misuse (2), acute suicidality, (2) or serious self-harm (2), experiencing a serious medical condition (2), had a neurodevelopmental condition (2) or learning disability (1). Other exclusion reasons included trauma (1), legal/court proceedings (1), and if the young person was commencing gender affirming hormone therapy or puberty blockers (1). In terms of the POMRF criteria ‘representativeness of the sample’, four studies scored good (Boggiss et al., 2020; Bratt et al., 2020; Joseph & Bance, 2020; Bluth et al., 2024). Three studies were scored fair (Khosravi et al., 2022; Damavandian et al., 2022; Lau-Zhu & Vella, 2023). Two studies were scored poor (Louis & Reyes, 2023; Tait, 2022) due to the lack of information on where participants were recruited from and their exclusion criteria, as this made it difficult to determine how representative the sample was of those seeking treatment. Overall, the exclusion criteria might have restricted the representativeness of some samples, as for example, many children who experience mental health difficulties and trauma are likely to also self-harm (Hu et al., 2017).

***Outcome measures***

**Compassion-focused measures.** Out of the nine included studies, seven used a specific compassion-focused outcome measure, whilst two studies did not (Louis & Reyes, 2023; Damavandian et al., 2022). The main compassion-focused outcome measure used was the Self-Compassion Scale (SCS). This scale was developed by Neff (2003) and comes in three formats: the full SCS, a short form (SCS-SF), and an adapted version for youth (SCS-Y). These are reliable and valid measures (Neff, 2016; Raes et al., 2011; Neff et al., 2021) which have been developed for specific age ranges. The SCS was developed for ages 14 and above, and the SCS-Y is designed for early adolescents aged 10-14. There were two studies that used the SCS, and both included participants younger than 14 years. Khosravi et al. (2022) used this measure despite the mean age of participants being 13, and Joseph and Bance (2020) also used this whilst having participants ranging from 12-17 years of age. Of the two studies that used the SCS-SF, Bratt et al. (2020) used this measure with an appropriate sample age range of 14-17, whilst Boggiss et al. (2020) used this despite having participants whose ages ranged from 12-16. The SCS-Y was used by two studies; Tait (2022) and Bluth et al. (2024) who both used this regardless of some of their samples being older than age 14 (range of 13-17).

There were two additional measures used. Lau-Zhu and Vella (2023) developed their own single visual analogue scale stating, ‘*I am kind to myself about my struggles’* which had a rating scale of ‘completely disagree’ to ‘completely agree’. Khosravi et al. (2022) used the Levels of Self-criticism Scale (Thompson & Zuroff, 2004), however, this measure has not been validated for use with children.

**Other measures.** Many other outcome measures were used by the nine studies to measure mental health domains and psychological constructs. There was much less consistency in the use of these measures between studies. Two studies (Bratt et al., 2020; Boggiss et al., 2020) used the Perceived Stress Scale (PSS; Cohen et al., 1983). Three studies had a common theme of measuring anxiety and/or depression (Lau-Zhu & Vella, 2023; Tait, 2022; Bluth et al., 2024). There were different tools used to measure this - the Revised Child Anxiety and Depression Scale (Chorpita et al.,2015), Depression, Anxiety and Stress Scale (DASS-21; Henry & Crawford, 2005), Pediatric Depression Scale (PROMIS; Kaat et al., 2020) and Depression Symptom Index–Suicidality Subscale (DSI‑SS; Metalsky & Joiner, 1997).

The reliability and validity of outcome measures used was variable across studies. There were five measures used which have not been validated for use with children and adolescents. One measure, the DASS (Henry & Crawford, 2005) has three constructs (depression, anxiety and stress) and has been found to not adequately differentiate between these in adolescent populations (Moore et al., 2017). The other four unvalidated measures were: Trauma-Related Shame Inventory (Øktedalen et al., 2014), Inventory of Statements About Self-Injury (Klonsky & Glenn, 2009), The Emotional Self-Regulation Questionnaire (Hofmann & Kashdan, 2010), The Aggression Questionnaire (Buss & Perry, 1992). Moreover, there were two measures used which have been validated for this population but were designed for a specific age range which the study sample did not fall within. The multidimensional Body-Self Relations Questionnaire (MBSRQ; Cash, 2000) was devised for adolescents aged 15 and above, but was used by Khosravi et al. (2022) whose sample had a mean age of 13. Additionally, the DSI‑SS (Metalsky & Joiner, 1997) has been validated for ages 15 and above, but was used by Bluth et al. (2024) whose sample included participants from age 13.

The POMRF item, ‘reliability and validity of outcome measures’ specifies that a study should be scored poorly if measures used fail to meet current standards of acceptability. Therefore, two studies (Boggiss et al., 2020; Damavandian et al., 2022) were scored as poor. In line with the POMRF criteria, if studies used some, but not all appropriate measures, they were scored as fair (Bluth et al., 2024; Joseph & Bance, 2020; Khosravi et al., 2022; Louis & Reyes, 2023; Lau-Zhu & Vella, 2023; Tait, 2022). Bratt et al. (2020) was the only study to score good as all of their outcome measures had good psychometric properties and were the best available to the authors.

***Intervention Format and Delivery***

The duration of the intervention was reported by all studies, although there was large variability in length. The shortest intervention was Boggiss et al. (2020) who held two 2.5-hour sessions, one week apart. The longest intervention duration was Tait (2022) whose study delivered 13 weekly 1-1.5-hour sessions. Of the nine included studies, two specified that they held the groups in person (Boggiss et al., 2020; Lau-Zhu & Vella, 2023), three online using Zoom (Tait, 2022; Louis & Reyes, 2023; Bluth et al., 2024), and four did not clearly report on the format of delivery (Khosravi et al.,2022; Bratt et al., 2020; Joseph & Bance, 2020; Damavandian et al., 2022).

In terms of therapist training and competence, there were four studies which did not record what therapist(s) delivered the intervention (Louis & Reyes, 2023; Damavandian et al., 2022; Tait, 2022; Joseph & Bance, 2020). Three studies, Lau-Zhu and Vella (2023), Bluth et al. (2024) and Khosravi et al. (2022) all utilised psychologists with appropriate training to deliver the intervention. Bratt et al. (2020)’s facilitator was a psychotherapist, and Boggiss et al. (2020)’s intervention was delivered by the author of the paper, who was supervised by a psychologist. Checks for adherence to the protocol and therapist competence were extremely limited across the studies. Six studies scored poorly on both treatment adherence and therapist competence (Tait, 2022; Lau-Zhu & Vella, 2023; Damavandian et al., 2022; Boggis et al, 2020; Bratt et al., 2020; Khosravi et al., 2022). Bluth et al. (2024) was the only study to score good for treatment adherence by providing adequate detail on how they considered this within their intervention. They used weekly checklists completed by a research assistant who sat in on the groups. Louis and Reyes (2023) scored fair, as they reported to use expert evaluation of the intervention, but the details around this were limited.

When evaluating the ‘manualised, replicable, specific treatment programs’ using the POMRF, there was considerable variability in the extent to which interventions were detailed and underpinned by empirical evidence. There were five studies which were scored as good for how they outlined their interventions (Joseph & Bance, 2020; Bluth et al., 2024; Khosravi et al., 2022; Lau-Zhu & Vella, 2023; Tait, 2022). Joseph and Bance (2020) delivered Compassion-Focused Visual Art Therapy and provided detailed descriptions of the session outlines. Bluth et al. (2024) used a mindful self-compassion program which drew upon the compassion work of Neff (2023). Khosravi et al. (2022) delivered CFT and referenced the work of Gilbert (2009). Lau-Zhu and Vella (2023) used the work of Gilbert (2014) to outline group sessions and provided a case conceptualisation. Tait (2022) used the work of Gilbert (2009) and recorded a detailed outline of session plans and activities used. Moreover, there were four studies which were rated as fair on the POMRF for their treatment programs (Bratt et al., 2020; Damavandian et al., 2022; Louis & Reyes, 2023; Boggiss et al., 2020). Bratt et al (2020) and Damavandian et al. (2022) referenced the work of Gilbert (2010), however, did not provide details of their session outlines. Louis and Reyes (2023) delivered cognitive self-compassion, and whilst they did outline the modules, there was limited reference to underlying psychological theory behind them. Boggiss et al. (2020) provided some, but limited intervention details on their brief self-compassion intervention.

***Study design***

There was some lack of clarity in the reporting of study design. Two studies described themselves as quasi experimental studies (Khosravi et al., 2022; Damavandian et al., 2022). Although, it was not clear whether Damavandian et al. (2022) used randomization and there were no details of a control group. Four studies used a within groups design where there was no control group (Lau-Zhu & Vella, 2023; Bluth et al., 2024; Tait, 2022; Louis & Reyes, 2023). Bluth et al. (2024)’s within groups design was used as part of a wider feasibility and acceptability study. There was one study which was an RCT (Joseph & Bance, 2020). Boggiss et al. (2020) described being a randomised waitlist-controlled feasibility trial and explained the challenge of being a fully powered trial due to their limited sample size. The remaining study by Bratt et al. (2020) was a between-groups design not utilising participant randomisation.

Four studies compared an experimental and control group (Khosravi et al., 2022; Bratt et al., 2020; Boggiss et al., 2020; Joseph & Bance, 2020). Bratt et al. (2020) used a treatment as usual (TAU) control group. The interventions provided in the TAU condition included cognitive behavioral therapy, systematic psychological treatments, clinical assessments, and psychosocial support. Those receiving TAU were offered between 1-14 sessions, whilst those in the experimental group were only offered 8, although the authors state there was no significant difference in number of sessions received between the groups. Two studies had a control group which received no treatment (Khosravi et al., 2022; Joseph & Bance, 2020). The remaining study by Boggiss et al. (2020) had a waitlist control group. Overall, studies that had a control group which received no treatment (Khosravi et al., 2022; Joseph & Bance, 2020) or a waitlist control group (Boggiss et al., 2020) scored poor on the POMRF for design. Studies with no control group are also scored poor, and this meant that a total of eight studies (Tait, 2022; Lau-Zhu & Vella, 2023; Damavandian et al., 2022; Boggiss et al., 2020; Louis & Reyes, 2023; Khosravi et al., 2022; Bluth et al., 2024; Joseph & Bance, 2020) scored low for this criterion.

***Statistical Data***

There were only two studies which used power calculation (Bratt et al., 2020; Damavandian et al., 2022). There were six studies which collected data pre- and post-group intervention (Joseph & Bance, 2020; Khosravi et al., 2022; Louis & Reyes, 2023; Boggiss et al., 2020; Lau-Zhu & Vella, 2023; Tait, 2022). The other three studies used a follow-up data point. Damavandian et al. (2022) had a one-month follow-up. Bluth et al. (2024) used a two-month follow-up, whilst Bratt et al. (2020) had the longest follow-up, which was six months post-group. However, the response rate was low, and they only reported on pre-post data.

Most studies scored good for their statistical analyses and presentation of results (Joseph & Bance, 2020; Bluth et al., 2024; Khosravi et al., 2022; Bratt et al., 2020; Louis & Reyes, 2023; Damavandian et al., 2022). There were two studies which were rated as fair. Boggiss et al. (2020) only reported on mean changes, and did not use any statistical tests, although they stated this was due to them being a feasibility study. Lau-Zhu and Vella (2023) report on outcome measure scores, but no group means, standard deviations or statistical tests. The criteria for good reporting of statistical data on the POMRF is ‘adequate statistical methods are used, and data are presented with M and SD’. However, it is important to note that whilst some studies met this criterion, there were four which did not report effect sizes of their results (Louis & Reyes, 2023; Boggiss et al., 2020; Lau-Zhu & Vella, 2023; Joseph & Bance, 2020). Thus, making it difficult to interpret the magnitude of the differences found.

**Study Outcomes**

Table 5 presents the mixed compassion and mental health outcomes. The table is organised by outcome variable, with studies within each category ordered by methodological quality (highest to lowest). There were three measures of effect size utilised. The most frequently used was Cohen’s d which has been interpreted using Cohen’s (1988) criteria: small effect (d= 0.2), medium effect (d = 0.5), large effect (d = 0.8 or greater). For studies using Hedges g, the criteria were: small effect (g = 0.2), medium effect (g = 0.5), and a large effect (g = 0.8). The third measure of effect used was partial eta squared. Cohen’s (1988) guidance was again used to interpret a small effect (n2 = 01), a medium effect (n2 = .06), and a large effect (n2 =.14 or greater).

***Self-compassion***

Self-compassion was measured in seven studies with varying results (Bluth et al., 2024; Bratt et al., 2020; Joseph & Bance, 2020; Boggiss et al., 2020; Lau-Zhu & Vella, 2023; Khosravi et al., 2022; Tait, 2022). Three studies reported significant improvements following the CFT intervention. In descending order of POMRF rating, Bluth et al. (2024), the highest rated study, found compassion increased at follow-up with a moderate-large effect size (d = 0.7). Joseph and Bance (2020) also recorded higher levels of self-compassion post-group but did not report on effect size. Khosravi et al. (2022) found a statistically significant increase in self-compassion with a large effect size (η2 = .345).

In contrast, four studies found no significant differences in self-compassion following their group intervention. In descending order, according to POMRF rating, Bratt et al. (2020), the highest rated study, found no significant differences between the CFT and TAU group. Boggiss et al. (2020) reported relatively small mean changes in compassion. Additionally, Lau-Zhu and Vella (2023) found that only two of their participants perceived their self-compassion to have increased post-group. Tait (2022) found no significant differences in compassion ratings.

One study measured levels of self-criticism alongside self-compassion (Khosravi et al., 2022). They compared a CFT group intervention to a no-treatment control group and found a statistically significant reduction in self-criticism following CFT. Therefore, engaging with CFT was associated with lower levels of self-criticism compared to receiving no treatment. This result had a large effect size (n2 = .363), indicating that group membership accounted for 36.3% of the variance in self-criticism scores.

***Stress***

Two studies used the PSS to measure the impact of a CFT group on stress. The highest rated study by Bratt et al. (2020) found no significant difference in stress ratings between the CFT and treatment as usual group. Similarly, Boggiss et al. (2020) found relatively small changes in stress levels following the CFT group, compared to a waitlist control.

***Anxiety and Depression***

Three studies investigated the impact of a CFT group on anxiety and depression ratings. Bluth et al. (2024), the highest rated study, found a reduction in depression ratings, but with a small effect size (d = - 0.19). Lau-Zhu and Vella (2023) found that five of their sample’s parents reported improvements in their child’s anxiety and low mood, and two children reported reductions in anxiety and depression. However, due to the limited data and statistical analysis provided in this study, these results should be viewed cautiously. Tait (2022) found no significant differences in anxiety and depression ratings post-group using the DASS. As the DASS is not validated for children and young people and has been found to not differentiate well between its three substructures of depression, anxiety, and stress (Moore et al., 2017), this may in part account for this finding.

***Other Outcomes***

There were other outcomes which were measured infrequently between the studies. Each of the following domains were only measured by one individual study, and so comparison across the literature was not possible. Bluth et al. (2024) found that suicidality of the participants decreased, and this had a large effect size (d = -0.99). They also investigated interpersonal needs within the transgender population and found that both burdensomeness and thwarted belongingness reduced at follow up. The effect sizes were small. Trauma-related shame was investigated by Joseph and Bance (2020) who found that when CFT was compared to no treatment, CFT led to lower levels of shame. The effect size was not recorded. Boggiss et al. (2020) conducted a paediatric study, and explored diabetes specific outcomes (disordered eating, diabetes distress, and self-care). They found small changes across all domains but did not report on the effect sizes of these changes. Tait (2022) investigated the impact on internalising and externalising problems and found no significant differences pre- to post-group. Damavandian et al. (2022) found that CFT led to a reduction in self-harming behaviours, and this had a large effect size (n2 = 0.918). They also measured emotion regulation and found that CFT was effective and had large effect sizes for two components of this, adaptive and tolerance, but a small effect size for the element of concealing. This study also measured aggression, anger, and hostility and found that CFT had a significant large effect on these three areas. Louis and Reyes (2023) found that self-esteem significantly improved; however, this should be interpreted cautiously as they did not report on the effect size.

**Supplementary File S2 -** Psychotherapy Outcome Study Methodology Rating Form (POMRF)

Note: If not enough information is given regarding a specific item a rating of 0 is given.

1. **Clarity of sample description**
2. Poor. Vague description of sample (e.g. only mentioned whether patients were diagnosed with the disorder).
3. Fair. Fair description of sample (e.g. mentioned inclusion/exclusion criteria, demographics, etc.).
4. Good. Good description of sample (e.g. mentioned inclusion/exclusion criteria, demographics, and the prevalence of comorbid disorders).
5. **Severity/chronicity of the disorder**
6. Poor. Severity/chronicity was not reported and/or subsyndromal patients were included in the sample.
7. Fair. All patients met the criteria for the disorder. Sample includes acute (>1yr) and/or low severity.
8. Good. Sample consisted entirely of chronic (41yr) patients of at least moderate severity.
9. **Representativeness of the sample**
10. Poor. Sample is very different from patients seeking treatment for the disorder (e.g. there are excessively strict exclusion criteria).
11. Fair. Sample is somewhat representative of patients seeking treatment for the disorder (e.g. patients were only excluded if they met criteria for other major disorders).
12. Good. Sample is very representative of patients seeking treatment for the disorder (e.g. authors made efforts to ensure representativeness of sample).
13. **Reliability of the diagnosis in question**
14. Poor. The diagnostic process was not reported, or not assessed with structured interviews by a trained interviewer.
15. Fair. The diagnosis was assessed with structured interview by a trained interviewer.
16. Good. The diagnosis was assessed with structured interview by a trained interviewer and adequate inter-rater reliability was demonstrated (e.g. kappa coefficient).
17. **Specificity of outcome measures**
18. Poor. Very broad outcome measures, not specific to the disorder (e.g. SCL-90R total score).
19. Fair. Moderately specific outcome measures.
20. Good. Specific outcome measures, such as a measure for each symptom cluster.
21. **Reliability and validity of outcome measures**
22. Poor. Measures have unknown psychometric properties, or properties that fail to meet current standards of acceptability.
23. Fair. Some, but not all measures have known or adequate psychometric properties.
24. Good. All measures have good psychometric properties. The outcome measures are the best available for the authors’ purpose
25. **Use of blind evaluators**
26. Poor. Blind assessor was not used (e.g. assessor was the therapist, assessor was not blind to treatment condition, or the authors do not specify).
27. Fair. Blind assessor was used, but no checks were used to assess the blind.
28. Good. Blind assessor was used in correct fashion. Checks were used to assess whether the assessor was aware of treatment condition.
29. **Assessor training**
30. Poor. Assessor training and accuracy are not specified, or are unacceptable.
31. Fair. Minimum criterion for assessor training is specified (e.g. assessor has had specific training in the use of the outcome measure), but accuracy is not monitored or reported.
32. Good. Minimum criterion of assessor training is specified. Inter-rater reliability was checked, and/or assessment procedures were calibrated during the study to prevent evaluator drift.
33. **Assignment to treatment**
34. Poor. Biased assignment, e.g. patients selected their own therapy or were assigned in another non-random fashion, or there is only one group.
35. Fair. Random or stratified assignment. There may be some systematic bias but not enough to pose a serious threat to internal validity. There may be therapist by treatment confounds. N may be too small to protect against bias.
36. Good. Randomor stratified assignment, and patients are randomly assigned to therapists within condition. When theoretically different treatments are used, each treatment is provided by a large enough number of different therapists. N is large enough to protect against bias.
37. **Design**
38. Poor. Active treatment vs. WLC, or briefly described TAU.
39. Fair. Active treatment vs. TAU with good description, or placebo condition.
40. Good. Active treatment vs. another previously empirically documented active treatment.
41. **Power analysis**
42. Poor. No power analysis was made prior to the initiation of the study.
43. Fair. A power analysis based on an estimated effect size was used.
44. Good. A data-informed power analysis was made and the sample size was decided accordingly.
45. **Assessment points**
46. Poor. Only pre- and post-treatment, or pre- and follow-up.
47. Fair. Pre-, post-, and follow-up <1 year.
48. Good. Pre-, post-, and follow-up >1 year.
49. **Manualized, replicable, specific treatment programs**
50. Poor. Description of treatment procedure is unclear, and treatment is not based on a publicly available, detailed treatment manual. Patients may be receiving multiple forms of treatment at once in an uncontrolled manner.
51. Fair. Treatment is not designed for the disorder, or description of the treatment is generally clear and based on a publicly available, detailed treatment manual, but there are some ambiguities about the procedure. Patients may have received additional forms of treatment, but this is balanced between groups or otherwise controlled.
52. Good. Treatment is designed for the disorder. A detailed treatment manual is available, and/or treatment is explained in sufficient detail for replication. No ambiguities about the treatment procedure. Patients receive only the treatment in question.
53. **Number of therapists**
54. Poor. Only one therapist, i.e. complete confounding between therapy and therapist.
55. Fair. At least two therapists, but the effect of therapist on outcome is not analyzed.
56. Good. Three, or more therapists, and the effect of therapist on outcome is analyzed.
57. **Therapist training/experience**
58. Poor. Very limited clinical experience of the treatment and/or disorder (e.g. students).
59. Fair. Some clinical experience of the treatment and/or disorder.
60. Good. Long clinical experience of the treatment and the disorder (e.g. practicing therapists)
61. **Checks for treatment adherence**
62. Poor. No checks were made to assure that the intervention was consistent with protocol.
63. Fair. Some checks were made (e.g. assessed a proportion of therapy tapes).
64. Good.Frequent checks were made (e.g. weekly supervision of each session using a detailed rating form).
65. **Checks for therapist competence**
66. Poor. No checks were made to assure that the intervention was delivered competently.
67. Fair. Some checks were made (e.g. assessed a proportion of therapy tapes).
68. Good.Frequent checks were made (e.g. weekly supervision of each session using a detailed rating form).
69. **Control of concomitant treatments (e.g. medications)**
70. Poor. No attempt to control for concomitant treatments, or no information about concomitant treatments provided. Patients may have been receiving other forms of treatment in addition to the study treatment.
71. Fair. Asked patients to keep medications stable and/or to discontinue other psychological therapies during the treatment.
72. Good. Ensured that patients did not receive any other treatments (medical or psychological) during the study.
73. **Handling of attrition**
74. Poor. Proportions of attrition are not described, or described but no dropout analysis is performed.
75. Fair. Proportions of attrition are described, and dropout analysis or intent-to-treat analysis is performed.
76. Good.No attrition, or proportions of attrition are described, dropout analysis is performed, and results are presented as intent-to-treat analysis.
77. **Statistical analyses and presentation of results**
78. Poor. Inadequate statistical methods are used and/or data are not fully presented.
79. Fair. Adequate statistical methods are used but data are not fully presented.
80. Good. Adequate statistical methods are used and data are presented with M and SD.
81. **Clinical significance**
82. Poor. No presentation of clinical significance was done.
83. Fair. An arbitrary criterion for clinical significance was used and the conditions were compared regarding percent clinically improved.
84. Good. Jacobson’s criteria for clinical significance were used and presented for a selection (or all) of the outcome measures, and conditions were compared regarding percent clinically improved.
85. **Equality of therapy hours (for non-WLC designs only)**
86. Poor. Conditions differ markedly (>20% difference in therapy hours).
87. Fair. Conditions differ somewhat (10–19% difference in therapy hours).
88. Good. Conditions do not differ (<10% difference in therapy hours).

**Supplementary File S3 -** POMRF Ratings for Included Studies

|  | Louis and Reyes (2023) | Damavandian et al. (2022) | Tait (2022) | Khosravi et al. (2022) | Lau-Zhu and Vella (2023) | Boggiss et al. (2020) | Joseph and Bance (2020) | Bratt et al. (2020) | Bluth et al. (2024) |
| --- | --- | --- | --- | --- | --- | --- | --- | --- | --- |
| Item 1 | Poor | Poor | Fair | Poor | Good | Good | Good | Good | Good |
| Item 2 |  |  |  |  |  |  |  |  |  |
| Item 3 | Poor | Fair | Poor | Fair | Fair | Fair | Good | Good | Good |
| Item 4 |  |  |  |  |  |  |  |  |  |
| Item 5 | Fair | Fair | Good | Good | Good | Good | Good | Good | Good |
| Item 6 | Fair | Poor | Fair | Fair | Fair | Poor | Fair | Good | Fair |
| Item 7 | Poor | Poor | Poor | Poor | Poor | Poor | Poor | Poor | Poor |
| Item 8 | Poor | Poor | Poor | Poor | Poor | Poor | Poor | Poor | Fair |
| Item 9 | Poor | Poor | Poor | Fair | Poor | Fair | Fair | Poor | Poor |
| Item 10 | Poor | Poor | Poor | Poor | Poor | Poor | Poor | Fair | Poor |
| Item 11 | Poor | Fair | Poor | Poor | Poor | Poor | Poor | Fair | Poor |
| Item 12 | Poor | Fair | Poor | Poor | Poor | Poor | Poor | Fair | Fair |
| Item 13 | Fair | Fair | Good | Good | Good | Fair | Good | Fair | Good |
| Item 14 | Poor | Poor | Poor | Fair | Fair | Fair | Fair | Fair | Fair |
| Item 15 | Poor | Poor | Poor | Good | Good | Good | Good | Good | Good |
| Item 16 | Fair | Poor | Poor | Poor | Poor | Poor | Fair | Poor | Good |
| Item 17 | Fair | Poor | Poor | Poor | Poor | Poor | Poor | Poor | Fair |
| Item 18 | Poor | Poor | Poor | Poor | Poor | Poor | Poor | Poor | Fair |
| Item 19 | Poor | Poor | Poor | Poor | Poor | Fair | Poor | Fair | Good |
| Item 20 | Good | Good | Good | Good | Fair | Fair | Good | Good | Good |
| Item 21 | Fair | Fair | Good | Fair | Good | Fair | Fair | Good | Fair |
| Item 22 | Poor | Poor | Fair | Poor | Poor | Poor | Poor | Poor | Poor |
